# Antimicrobial susceptibility and multilocus sequence typing of *Mycoplasma capricolum* subsp. *capricolum*

**DOI:** 10.1371/journal.pone.0174700

**Published:** 2017-03-27

**Authors:** Juan Tatay-Dualde, Miranda Prats-van der Ham, Christian de la Fe, Ana Paterna, Antonio Sánchez, Juan Carlos Corrales, Antonio Contreras, Sebastiana Tola, Ángel Gómez-Martin

**Affiliations:** 1 Department of Animal Health, Faculty of Veterinary Sciences, Regional Campus of International Excellence “Campus Mare Nostrum”, Universidad de Murcia, Campus de Espinardo s/n., Murcia, Spain; 2 Istituto Zooprofilattico Sperimentale della Sardegna “G. Pegreffi”, Sassari, Italy; Miami University, UNITED STATES

## Abstract

*Mycoplasma capricolum* subsp. *capricolum* is one of the causative agents of contagious agalactia (CA). Nevertheless, there is still a lack of information about its antimicrobial susceptibility and genetic characteristics. Therefore, the aim of this work was to study the antimicrobial and genetic variability of different *Mycoplasma capricolum* subsp. *capricolum* field isolates. For this purpose, the growth inhibition effect of 18 antimicrobials and a multilocus sequence typing (MLST) scheme based on five housekeeping genes (*fusA*, *glpQ*, *gyrB*, *lepA* and *rpoB*) were performed on 32 selected field isolates from Italy and Spain.The results showed a wide range of growth inhibitory effects for almost all the antimicrobials studied. Macrolides presented lower efficacy inhibiting Mcc growth than in previous works performed on other CA-causative mycoplasmas. Erythromycin was not able to inhibit the growth of any of the studied strains, contrary to doxycycline, which inhibited the growth of all of them from low concentrations. On the other hand, the study of the concatenated genes revealed a high genetic variability among the different Mcc isolates. Hence, these genetic variations were greater than the ones reported in prior works on other mycoplasma species.

## Introduction

*Mycoplasma capricolum* subsp. c*apricolum* (Mcc) is one of the causative agents of contagious agalactia (CA) [1]. In some regions such as France [2] or the Canary Islands (Spain) it is the second most frequently isolated CA-causing agent, and its presence has been related with severe outbreaks of this disease [3]. Furthermore, Mcc has been recently detected in a human case in which the patient displayed symptoms of septicaemia [4, 5].

Antimicrobial therapy is one of the main tools used to control mycoplasmoses, including CA. Thus, macrolides, fluoroquinolones and tetracyclines are widely employed in CA endemic areas [6]. In this context, it is noticeable that the frequent use of these antimicrobials has been related with an increase of *in vitro* antimicrobial resistance of the bovine pathogen *M*. *bovis* [7]. Regarding CA, previous studies have described antimicrobial resistances on field isolates of *Mycoplasma* (*M*.) *agalactie*, *M*. *mycoides* subsp. *capri* and *M*. *putrefaciens* [8–11]. Notwithstanding, there is a lack of information concerning the antimicrobial susceptibility of Mcc, which has only been assessed in 8 field isolates against 6 antimicrobials [12].

Regarding the control of mycoplasmoses, the assessment of the genetic diversity among different isolates has proved to be useful to epidemiological studies, by means of which infectious diseases are monitored. For this purpose, several typing techniques have been developed so as to compare isolates, including multilocus sequence typing (MLST). This technique utilizes sequences of internal fragments from housekeeping genes in order to characterize bacterial isolates, and it seems to be the most appropriate technique to analyze evolutionary relationships amongst mycoplasma isolates [13–16]. Hence, in previous studies with other mycoplasmas, a great genetic diversity between isolates from the same area (country) [13, 17] or even from the same farm [16] was observed. As for the members of the *M*. *mycoides* cluster, to which Mcc belongs, a scheme based on five housekeeping genes (*fusA*, *glpQ*, *gyrB*, *lepA* and *rpoB*) has been described [18]. Nevertheless, apart from the isolates analyzed in that study [18], there are no previous works in which this technique has been used to analyze the genetic diversity of different Mcc field isolates.

Taking into account the previously described facts, the aim of the present study was to characterize different Mcc isolates from a CA-endemic area by antimicrobial susceptibility testing and molecular typing in order to determine the suitability of different antimicrobial treatments against this pathogen and its genetic profile, which affects the monitoring and control of this disease.

## Material and methods

### Mycoplasma isolates

32 Mcc isolates were investigated, including the reference strain of Mcc, California Kid (CK) (NCTC 10154). Epidemiological data of the studied isolates and CK are shown in **Table 1.** All isolates were identified by previously described PCR protocols targeting the genes *glk* and *fusA* [18, 19].

**Table 1:** Description of the strains studied.

Strain	Origin (Province, Region, Country)	Host	Sample	Year
**CK (NCTC 10154)**	USA	Goat	Arthritis	1955
**Cap1**	Spain, Murcia.	Goat	Auricular swab	2009
**Cap8**	Spain, Murcia	Goat	Auricular swab	2011
Cap9**	Spain, Murcia	Goat	Auricular swab	2011
Cap10**	Spain, Murcia	Goat	Auricular swab	2011
**Cap3**	Spain, Andalusia	Goat	Mastitic milk	2011
**Cap4**	Spain, Andalusia	Goat	Auricular swab	2009
**Cap6**	Spain, Andalusia	Goat	Auricular swab	2009
**Cap7**	Spain, Andalusia	Goat	Auricular swab	2009
**Cap2**	Spain, Canary Islands, Gran Canaria	Goat	Auricular swab	2009
**Cap15**	Spain, Canary Islands, La Palma	Goat	Auricular swab	2015
Cap16*	Spain, Canary Islands, Lanzarote	Goat	Mastitic milk	2015
Cap17*	Spain, Canary Islands, Lanzarote	Goat	Mastitic milk	2015
Cap18*	Spain, Canary Islands, Lanzarote	Goat	Mastitic milk	2015
Cap19*	Spain, Canary Islands, Lanzarote	Goat	Mastitic milk	2015
Cap20*	Spain, Canary Islands, Lanzarote	Goat	Mastitic milk	2015
**Cap21**	Spain, Canary Islands, Lanzarote	Goat	Auricular swab	2015
Cap22*	Spain, Canary Islands, Lanzarote	Goat	Mastitic milk	2015
**Cap23**	Spain, Canary Islands, Lanzarote	Goat	Mastitic milk	2015
**Cap24**	Spain, Canary Islands, Lanzarote	Goat	Mastitic milk	2015
Cap25*	Spain, Canary Islands, Lanzarote	Goat	Mastitic milk	2015
**874**	Italy, Sardinia	Sheep	Lung	2007
6721***	Italy, Sardinia	Goat	Brain	2007
**26909**	Italy, Sardinia	Goat	Skin abscess	2005
**20413**	Italy, Sardinia	Goat	Mastitic milk	2013
78106***	Italy, Sardinia	Goat	Lung	2006
**87194**	Italy, Sardinia	Sheep	Brain	2015
**95748**	Italy, Sardinia	Goat	Milk	2015
**30666**	Italy, Sardinia	Goat	Milk	2016
**26918**	Italy, Sardinia	Sheep	Joint fluid	2014
**54731**	Italy, Sardinia	Sheep	Joint fluid	2011
**68873**	Italy, Sardinia	Sheep	Brain	2012

*, **, ***: Isolates from the same herd at different time points

### Microdilution susceptibility test

For the antimicrobial susceptibility tests, Mcc isolates were grown in PPLO medium (Difco, France), supplemented with Equine Serum (HyClone, USA) without any antimicrobial [20], during 24 hours until reached the stationary growing phase. Subsequently, the concentration of the inocula was determined by the simple quantification method of viable mycoplasmas [21], and then diluted with sterile saline solution up to the desired concentration.

Antimicrobial effect was assessed by determining the minimum inhibitory concentration (MIC), following previously described recommendations [22]. We evaluated the effect of 18 antimicrobials: spectinomycin (Sigma-Aldrich, USA), kanamycin (Sigma-Aldrich, USA), gentamycin (Sigma-Aldrich, USA), neomycin (Sigma-Aldrich, USA) and streptomycin (Sigma-Aldrich, USA) in a range from 128 μg/ml to 16 μg/ml; ciprofloxacin (Fluka, USA), enrofloxacin (Fluka, USA), marbofloxacin (Tokyo Chemical Industry, Japan), danofloxacin (Fluka, USA), moxifloxacin (Cayman Chemical Company, USA), norfloxacin (Sigma-Aldrich, USA), doxycycline (Sigma-Aldrich, USA), tylosin (Fluka, USA) and tilmicosin (Molekula, UK), which were studied in a range from 12.8 μg/ml to 0.006 μg/ml; and erythromycin (Fluka, USA), spiramycin (Sigma-Aldrich, USA), clindamycin (Cayman Chemical Company, USA) and lincomycin (Sigma-Aldrich, USA), which were evaluated in a range from 12.8 μg/ml to 0.8 μg/ml. The concentration range of these antimicrobials was selected considering data published in previous studies [8–10]. Different concentrations of antimicrobials were obtained by serial two-fold dilutions from the highest to the lowest concentration.

96-wells microtitre plates were used to carry out this method. In each well 150 μl of PH medium with 0.007% of phenol red, 25.6 μl of each antimicrobial dilution and 25 μl of the diluted inocula were added in order to reach a concentration of 10^3^−10^5^ CFU/ml. Furthermore, a well without antimicrobial was used as a positive control and a well without neither antimicrobial nor inoculum as a negative control. Plates were incubated at 37°C in a humid atmosphere with 5% CO_2_ and they were read when a change of color appeared in the positive control due to the acidification of the medium. These analyses were performed in duplicates. MIC is defined as the minimum concentration of antimicrobial at which no growth is observed. Since there are no established breakpoints for MIC in Mcc, results were evaluated according to the breakpoints reported for other mycoplasma species [23, 24] or by the European Committee on Antimicrobial Susceptibility Testing [25] **(Table 2)**. Furthermore, the antimicrobial concentration at which the 50% of strains were inhibited (MIC_50_) and the concentration at which the 90% of strains were inhibited (MIC_90_) were also calculated.

**Table 2:** Minimal inhibitory concentration (MIC) range, MIC_50,_ MIC_90_ for *Mycoplasma capricolum* subsp. c*apricolum* field isolates for 18 antimicrobials and their MIC against the reference strain.

	MIC range (μg/ml)	MIC_50_ (μg/ml)	MIC_90_ (μg/ml)	California Kid (μg/ml)	Breakpoints; susceptible, intermediate, resistant (μg/ml) [Reference]	Non susceptible strains (%)*
**Spectinomycin**	16->128	32	>128	>128	≤32, 64, ≥128 [24]	64.5
**Kanamycin**	16->128	32	>128	>128	ND	
**Gentamycin**	3.2->128	64	>128	>128	ND	
**Neomycin**	32->128	>128	>128	>128	ND	
**Streptomycin**	16->128	>128	>128	>128	ND	
**Ciprofloxacin**	0.01->12.8	0.2	1.6	0.1	≤ 0.5, ND, ≥1 [25]	12.9
**Enrofloxacin**	0.025–3.2	0.2	0.4	0.05	≤ 0.5, 1, ≥2 [23]	6.5
**Marbofloxacin**	0.1–12.8	0.4	0.4	0.05	ND	
**Danofloxacin**	0.1–6.4	0.1	0.4	0.1	≤ 0.25, ND, ND [24]	12.9
**Moxifloxacin**	0.006–0.8	0.05	0.1	0.025	≤ 0.25, ND, ≥0.5 [26]	6.7
**Norfloxacin**	0.4->12.8	0.8	1.6	0.8	≤ 0.5, ND, ≥1 [25]	77.4
**Doxycycline**	0.01–0.2	0.05	0.1	0.01	ND	
**Tylosin**	0.05->12.8	0.1	>12.8	0.01	≤1, 2, ≥4 [23]	19.4
**Tilmicosin**	0.01->128	0.025	>12.8	0.01	≤8, 16, ≥32 [24]	12.9
**Erythromycin**	3.2->12.8	>12.8	>12.8	>12.8	≤ 0.5, ND, ≥1 [26]	100
**Spiramycin**	3.2->12.8	>12.8	>12.8	6.4	ND	
**Clindamycin**	0.01->12.8	0.2	>12.8	0.01	≤ 0.25, ND, ≥ 0.5 [26]	48.4
**Lincomycin**	0.2->12.8	0.8	12.8	0.2	ND	

ND: No data

*: intermediate + resistant strains

### Multilocus sequence typing

Mycoplasmal genomic DNA was obtained using a classical phenol-chloroform extraction from 25 ml volume of broth culture of each Mcc isolate [27].

The MLST study was based on the previously developed scheme for the *M*. *mycoides* cluster typing [18]. Hence, the five housekeeping genes proposed in that study (*fusA*, *glpQ*, *gyrB*, *lepA* and *rpoB*) were analyzed. PCRs were performed with Phusion High-Fidelity PCR Kit (Thermo Scientific, USA). They were done in a final volume of 25 μl containing 0.5 μM of each primer, 0.1 mM dNTPs, 1X Phusion Buffer and 0.02 units of Phusion DNA Polymerase. Amplifications were performed applying the following incubation conditions: one cycle of 95°C for 30 s; 35 cycles of 95°C for 10 s, variable annealing temperatures (detailed in **Table 3**) for 1 min, 72°C for 1 min and a final incubation at 72°C for 10 min. These reactions were performed in an i-Cycler (Biorad, USA) thermal cycler. PCR products were subjected to electrophoresis in 1% agarose gels containing 0.005% of RedSafe (iNtRON Biotechnology, Korea) DNA staining, and visualized under UV light. Subsequently, PCR products were purified using QIAquick PCR Purification Kits (Qiagen, The Netherlands). After that, PCR products were sequenced at the molecular biology service of the University of Murcia and compared in both forward and reverse directions with the same primers as used for the PCR. The sequences obtained in this study for each MLST loci were trimmed to the same size as previously described (16), and are detailed in **S1 Table**. Moreover, the sequences of these five loci from the reference strain for Mcc, California Kid (CP000123), were retrieved from GenBank.

**Table 3:** Multilocus sequence typing (MLST) loci, PCR details and variability of each locus.

Loci	Annealing T (°C)	Amplicon (bp)	Squence analyzed (bp)	Variable sites	Informative sites
***fusA***	59	781	746	13	9
***glpQ***	58.2	695	664	9	8
***gyrB***	63.1	635	609	21	14
***lepA***	59	1097	912	29	21
***rpoB***	64.8	824	795	20	19
**Concatenated set**	NA	NA	3726	92	71

Sequence analyzes were conducted using MEGA6 [28]. Pairwise distances were calculated as the proportion of non-matching sites between pairs of sequences. Phylogenetic trees were built using the neighbor-joining algorithm based on pairwise dissimilarities between strains [29]. The ‘‘pairwise gap block correction” option was selected with a ‘‘minimal length for gap blocks” of 1 nucleotide so that all consecutive gaps were considered a single event. Bootstrap analyses with 1000 replicates were performed.

## Results

### Antimicrobial MICs

MIC range, MIC_50_, MIC_90_ and MIC values for the studied field isolates and the reference strain of Mcc are provided in **Table 2.** Moreover, MIC values for each of the studied strains are shown in S2 Table.

All the Mcc isolates showed high MIC values for the studied aminoglycosides. Hence, spectinomycin, kanamycin and streptomycin returned minimum MIC values of at least 16 μg/ml, whereas gentamycin and neomycin yielded 32 μg/ml. Moreover, the lowest MIC_50_ value obtained was 32 μg/ml and the MIC_90_ values were over 128 μg/ml.

On the other hand, the MIC_50_ values obtained for quinolones were lower than 0.4 μg/ml, except for norfloxacin which showed the highest value (0.8 μg/ml) within this group. MIC_90_ values were also lower than 0.5 μg/ml, except for ciprofloxacin (1.6 μg/ml) and norfloxacin (1.6 μg/ml). However, 2 field isolates presented MIC values over 1 μg/ml for all quinolones except for moxifloxacin. Besides, 2 other isolates were resistant only to ciprofloxacin (>12.8 μg/ml) and 24 of the studied isolates showed high MIC values (> 0.5 μg/ml) for norfloxacin.

Doxycycline was the antimicrobial which showed the lowest MIC results in all the studied parameters (MIC range, MIC_50_ and MIC_90_). Therefore, no resistant strains to this antimicrobial group were found.

Finally, the group of macrolides and lincosamides showed great variations between the different antimicrobials assessed. Tylosin and tilmicosin were the most effective ones within this group. However, 6 isolates returned high MIC values (> 1 μg/ml) for tylosin and 4 for tilmicosin (24). Furthermore, the MIC_90_ for both antimicrobials was >12 μg/ml. On the other hand, clindamycin and lincomycin showed a great diversity of values for the different isolates studied, with a MIC_50_ of 0.2 and 0.8 μg/ml, respectively, and a MIC_90_ >12.8 for both antimicrobials. Erythromycin was not effective to inhibit the growth of any of the assessed isolates of Mcc (MIC >12.8 μg/ml), except for one which MIC was 3.2 μg/ml.

### MLST analysis

The sequences of five housekeeping genes were analyzed, comprising a total of 3726 base pairs. Twenty five Mcc isolates were partially sequenced and compared to the CK strain, which data were retrieved from the GenBank database (CP 000123). All these sequences were aligned and trimmed to the same size, providing gene fragments ranging from 609 to 912 bp (**Table 3**), and their GenBank entrance numbers are available at the **S1 Table**. The number of single nucleotide polymorphisms (SNP) observed within each individual locus as well as in the concatenated sequences of the five loci are indicated in **Table 3**. Thus, from a total of 3726 base pairs, 92 were variable sites and 71 were informative sites.

**Fig 1:**
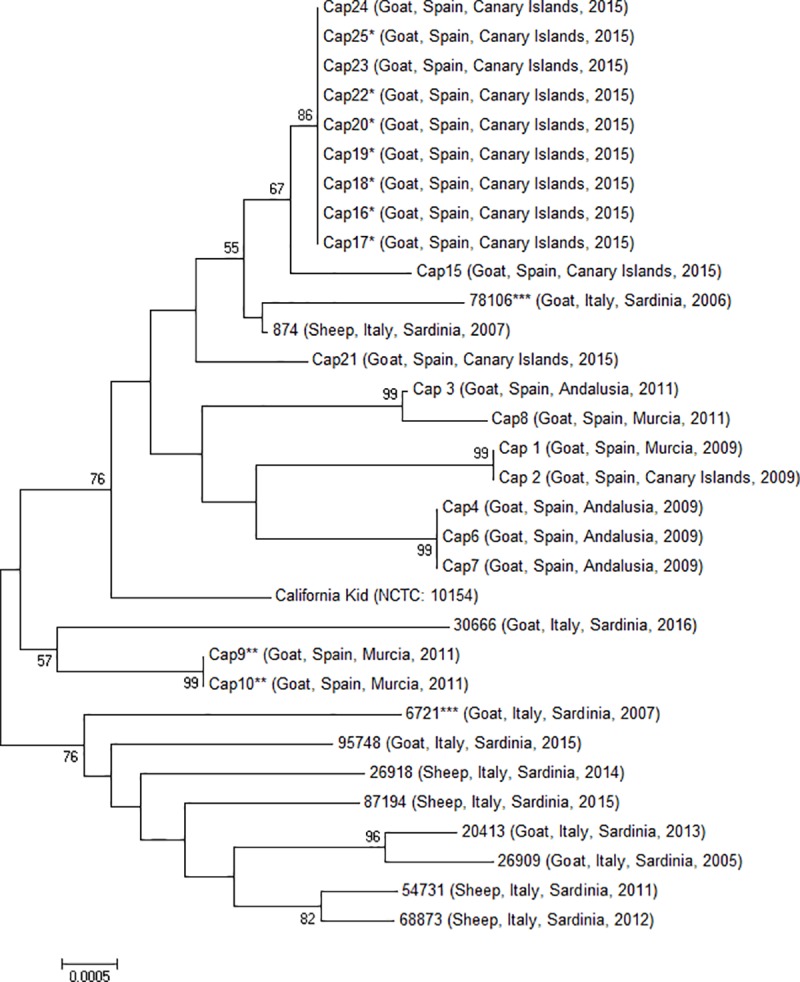
Phylogenetic tree inferred from the concatenated sequences of the five housekeeping genes, corresponding to the 31 field isolates analyzed in this study and California Kid strain. The tree was constructed using the neighbor-joining algorithm. Bootstrap percentage values were calculated from 1000 resamplings and values over 50% are displayed. *, **, ***: Isolates from the same herd.

Regarding the study of the five concatenated sequences, pairwise distance analyses are shown in **S1 Fig**. The maximum divergence observed between the studied isolates is 0.9%, and the mean intra-group distance was 0.5%. Phylogenetic analysis using the neighbor-joining method on the concatenated sequences is displayed in **Fig 1**. Hence, the resultant phylogenetic tree did not show any apparent correlation between the phylogenic origin of the strains and their anatomic source (auricular swab or mastitic milk) or their isolation date. Besides, there were not reported phylogenetic differences between sheep and goat isolates. In fact, both groups of strains shared their phylogenetic origin and showed the same genetic variability. Nevertheless, the majority of field isolates coming from Italy were visibly separated from the other strains (bootstrap value of 76%). However, the 78106 and 874 isolates which were from Italy too were clearly separated from this group, being most related to isolates from the Canary Islands (bootstrap 55%). Additionally, whilst all the Spanish isolates from 2015 appeared next to each other in the resulting phylogenetic tree, this kind of correlation could not observed between the isolates from other years.

Since previous studies reported phylogenetic differences between isolates coming from the same farms [16, 17], our study included the study of three farms from which more than one isolate were retrieved. Phylogenetic differences appeared between different isolates coming from one of these farms, but only when isolates were retrieved at different years.

Concerning individual gene study, the phylogenetic trees based on single locus sequences are shown in **S2–S6 Figs**. Thus, the tree based on *fusA* sequences (**S2 Fig**) showed three main branches, one of which grouped the Italian isolates, similarly to the concatenated tree. Evolutionary distance trees based on *glpQ* and *gyrB* trees (**S3** and **S4 Figs**) were incongruent with the phylogeny based on concatenated sequences. The tree based on *lepA* sequences (**S5 Fig**) showed two main branches. One of them could be related to the first branches displayed on the concatenated genes tree (**Fig 1**) (until the California Kid branch), and the other main branch might be associated with the last divisions of this tree. Last, the study of the *rpoB* sequences (**S6 Fig**) resulted in a tree with three main branches. One of them could be related to the Italian isolates, and another branch was able to group the isolates from the Canary Islands together with strain 874 (Italy), in the same way as it occurred in the concatenated genes tree.

## Discussion

Antimicrobial MIC studies are an essential tool to evaluate mycoplasmal antimicrobial resistances [22], and have also proved to be useful to monitor their evolution over time as the massive use of antimicrobials in infected flocks has been related with an increase of *in vitro* resistances [7]. Besides, to our knowledge, this is the first phylogenetic analysis performed on Mcc since an MLST scheme for the *M*. *mycoides* cluster was developed [18]. In this sense, epidemiological investigations, which are essential for disease surveillance and control, need suitable typing tools to carry out phylogenetic studies with enough resolution. Hence, sequenced-based typing methods have demonstrated to be technically simple, robust and portable [30].

In the present study, enrofloxacin, marbofloxacin, danofloxacin and moxifloxacin (fluoroquinolones), and doxycycline (tetracycline) were the most effective antimicrobials inhibiting the growth of the different Mcc isolates. Thus, these antimicrobials showed similar inhibitory concentrations as reported in previous studies carried out on other CA-causing mycoplasmas [8, 9, 11, 12]. Concerning the susceptibility breakpoints proposed in previous works on different mycoplasma species [23, 24] (**Table 2**), two of our isolates must be considered as resistant to enrofloxacin. Moreover, since the proposed breakpoint for danofloxacin was 0.25 μg/ml [24], four of the studied isolates must be also considered as resistant to this antimicrobial.

Our study exhibits a high value of MIC_90_ for ciprofloxacin and norfloxacin. In this sense, 4 isolates showed high MIC values for ciprofloxacin and 2 isolates (Cap20 and Cap23) yielded MIC values higher than 1 μg/ml for all the studied quinolones. Therefore, doxycycline is the only antimicrobial effective in inhibiting the growth of Mcc at low concentrations for all the assessed isolates. These data, together with the available pharmacokinetic studies [31] and successful clinical applications of this antimibrobial [5], highlight the advantages of using doxycycline to effectively treat Mcc infections.

Regarding macrolides and lincosamides, taking into consideration the proposed susceptibility breakpoints for tylosin and clindamycin [23, 32], 6 of our isolates were resistant to tylosin and 9 were resistant to clindamycin. These results are in contrast with prior data obtained for *M*. *agalactiae****)***[9], where clindamycin was the most effective antimicrobial analyzed. In the present work, MIC ranges for tylosin and tilmicosin were wide and thus, their MIC_90_ values were >12.8 μg/ml. These values were noticeably higher than the values shown in previous studies [8, 9]. Besides, the MIC values obtained for erythromycin and spiramycin were also higher than the outcomes of previous studies on other members of the *M*. *mycoides* cluster [8, 12]. These remarkable differences could be explained by the extensive use of macrolides such as tylosin and erythromycin to treat CA [6], explaining why Mcc strains retrieved between years 2003 and 2004 were more sensitive to antimicrobials [12] than the isolates assessed in this work, as it has been proved in other mycoplasma species such as *M*. *bovis* [7]. In fact, when comparing the MIC results of CK with those reported for the rest of the studied field isolates, CK always showed equal or lower MIC values than the MIC_50_ value for each antimicrobial. This finding highlights an increase of MICs over time, as CK is an elder strain.

None of the assessed aminoglycosides was able to inhibit the growth of any Mcc isolate with a concentration lower than 16 μg/ml. Moreover, their MIC_50_ and MIC_90_ values showed that these antimicrobials are not effective against this pathogen. These results are in agreement with those described in previous studies performed on other mycoplasma species [8, 10, 11, 33]. Hence, our work supports the previously proposed hypothesis about an intrinsic resistance of the mycoplasmas of the *M*. *mycoides* cluster to aminoglycosides [8].

Regarding the MIC results together with the MLST analysis, as exposed in S2 Table, our study showed that epidemiologically related strains did not always present similar MIC values. In fact, some of the strains belonging to the main phylogenetic group (Cap24, Cap25, Cap23, Cap22, Cap20, Cap19, Cap18, Cap16 and Cap17), showed low MICs for macrolides, whereas other isolates with the same phylogenetic profile proved to be resistant to these antimicrobials. Hence, our results are more in concordance with those obtained in previous studies with M. agalactiae [9] than those reported for M. bovis [34], in which molecular tying results and MIC values were closely related.

One of the most remarkable results of the present phylogenetic study was the great genetic variability showed between the 32 Mcc strains assessed. Although our mean intra-group distance was lower than in previous studies conducted on other mycoplasma species [15], there were only two groups of indistinguishable strains which had not been isolated from the same herds (Cap4, Cap6 and Cap7; Cap1 and Cap2). Hence, the rest of the isolates showed significant differences in at least one of the studied genes, contrary to the genetic homogeneity reported for *M*. *agalactiae* strains also isolated in Spain [35]. In this context, the genetic variability showed by Mcc at the present work is in concordance with a prior work with *Mycoplasma mycoides* subsp. *capri* [17] which is closely related to Mcc and also reported a substantial genetic diversity. This fact could be explained by the proved genetic transmission which happens amongst different mycoplasma species when they coinfect a single host [36]. In this sense, Mcc and *Mycoplasma mycoides* subsp. *capri* are mainly isolated from goats, which may be infected by more than one CA-causing mycoplasma species, as opposed to sheep, which are mainly affected by *M*. *agalactiae*. Therefore, goats have shown to be prone to mixed mycoplasmal infections [1], where horizontal gene transfer has demonstrated to effectively contribute to individual evolutionary differences.

Moreover, our results also suggested a correlation between the genetic profile and geographical origin of the studied strains, which has not been described for other mycoplasma species such as *M*. *agalactiae* [13]. Thus, our phylogenetic tree revealed a common branch for the majority of Italian isolates, which were clearly different from the Spanish isolates. Nevertheless, although a certain evolutionary approach was observed in the isolates from the Canary Islands (except for Cap2), it was not possible to discern a clear correlation between the evolutionary distances and geographical origin between the Spanish field isolates. This could be due to the fact that Mcc strains from the mainland (Iberian Peninsula) might have evolved together, while clonal expansion to more distant territories such as the Canary Islands or Italy may be less evident, resulting in greater similarities between isolates from these areas. However, despite the relationship suggested in this study between the geographical origin (Canary Islands, Italy and the Spanish mainland) and genetic evolution, further studies analyzing more isolates are necessary to gain a better understanding of the evolution of this pathogen in CA-endemic areas.

In connection with previous studies performed on other mycoplasma species which MLST schemes showed genetic diversity between isolates coming from the same flocks [16, 17], in the present work, different strains isolated from the same herds were selected in order to compare them. In this context, when isolates were taken at different years, phylogenetic differences were shown, highlighting the genetic variability of this pathogen. However, no differences were observed in any of the genes studied among isolates which had been obtained from the same herds during the same year. This result is contrary to the outcomes reported for *M*. *arginini* [16] and *Mycoplasma mycoides* subsp. *capri* [17], which showed significant differences between the MLST analyses of different strains isolated in the same conditions. Nevertheless, those studies [16, 17] analyzed a greater number of herds with multiple isolates and therefore, further studies are necessary to determine if MLST analysis is also able to demonstrate genetic diversity in Mcc isolates within farms, as suggested by our results.

Last, the individual study of *fusA*, *glpQ* and *gyrB* showed that these genes presented less informative sites than the other two loci assessed (**Table 3**), which differs from the results previously reported for *glpQ* and *gyrB* [18]. However, this fact could explain why the phylogenetic trees of *glpQ* and *gyrB* (**S3** and **S4 Figs**) are less consistent with the concatenated genes tree. Accordingly to the outcomes of the MLST scheme for the *M*. *mycoides* cluster [18], in our study the *fusA* gene showed the least informative sites but its phylogenetic tree (**S2 Fig**) managed to partly differentiate the main groups displayed at the concatenated genes tree. However, since *lepA* and *rpoB* were the genes with the most informative sites, their phylogenetic trees (**S5** and **S6 Figs**) were capable of distinguishing more branches in concordance with the joint study of the five housekeeping genes. Therefore, these genes are the ones providing more useful information when performing genetic typing analysis of Mcc. Thus, they should be chosen when only one gene is to be studied.

## Conclusions

Doxycycline was the most effective antimicrobial inhibiting Mcc field isolates *in vitro*; therefore, it should be one of the antimicrobials of choice to treat CA caused by Mcc. Wide MIC ranges and the increment of MIC values observed in comparison with previous studies emphasize the importance of analyzing each isolate individually so as to select the most adequate antimicrobial in each case.

The MLST scheme applied in the present study confirms its suitability to assess the genetic variability of Mcc isolates from separately locations within a CA-endemic area. Moreover, the great genetic diversity observed in this work between the different isolates studied suggests that Mcc could present higher genetic diversity than other mycoplasma species.

## Supporting information

S1 Fig Pairwaise distance analysis of concatenated protein-coding sequences from 32 strains calculated as the proportion of non-matching sites between pairs of sequences. (PDF)

S2 Fig Evolutionary tree based on *fusA* sequences. The tree was constructed using the neighbor-joining algorithm. Bootstrap percentage values were calculated from 1000 replications and values over 50% are displayed.
(PDF)

S3 Fig Evolutionary tree based on *glpQ* sequences. The tree was constructed using the neighbor-joining algorithm. Bootstrap percentage values were calculated from 1000 replications and values over 50% are displayed.
(PDF)

S4 Fig Evolutionary tree based on *gyrB* sequences. The tree was constructed using the neighbor-joining algorithm. Bootstrap percentage values were calculated from 1000 replications and values over 50% are displayed.
(PDF)

S5 Fig Evolutionary tree based on *lepA* sequences. The tree was constructed using the neighbor-joining algorithm. Bootstrap percentage values were calculated from 1000 replications and values over 50% are displayed.
(PDF)

S6 Fig Evolutionary tree based on *rpoB* sequences. The tree was constructed using the neighbor-joining algorithm. Bootstrap percentage values were calculated from 1000 replications and values over 50% are displayed.
(PDF)

S1 Table GenBank accession numbers of sequences obtained used in this study. (PDF)

S2 Table MIC results for each studied strain. (PDF)
