# Supplementary material for: Antimicrobial susceptibility and multilocus sequence typing of Mycoplasma capricolum subsp. capricolum
Source: PLoS One. 2017 Mar 27;12(3):e0174700. doi: 10.1371/journal.pone.0174700 (PMC5367824; doi:10.1371/journal.pone.0174700)
Supplement: S1 Fig — (PDF) [file pone.0174700.s001.pdf]

S1 Fig.

Pairwise distance analysis of concatenated protein-coding sequences from 32 strains calculated as the proportion of non-matching sites between pairs of sequences.

	1	2	3	4	5	6	7	8	9	10	11	12	13	14	15	16	17	18	19	20	21	22	23	24	25	26	27	28	29	30	31
1. 20413																															
2. 78106	0,005																														
3. Cap17	0,007	0,003																													
4. 87194	0,004	0,006	0,006																												
5. 95748	0,005	0,006	0,006	0,004																											
6. 30666	0,008	0,008	0,007	0,007	0,007																										
7. Cap1	0,008	0,006	0,005	0,007	0,007	0,007																									
8. CK	0,007	0,004	0,003	0,005	0,005	0,006	0,005																								
9. Cap2	0,008	0,006	0,005	0,007	0,007	0,007	0,000	0,005																							
10. Cap3	0,008	0,005	0,004	0,008	0,007	0,007	0,005	0,004	0,005																						
11. Cap4	0,009	0,005	0,004	0,008	0,007	0,008	0,004	0,005	0,004	0,004																					
12. Cap6	0,009	0,005	0,004	0,008	0,007	0,008	0,004	0,005	0,004	0,004	0,000																				
13. Cap7	0,009	0,005	0,004	0,008	0,007	0,008	0,004	0,005	0,004	0,004	0,000	0,000																			
14. Cap8	0,008	0,005	0,005	0,008	0,007	0,008	0,006	0,005	0,006	0,001	0,004	0,004	0,004																		
15. Cap9	0,006	0,005	0,005	0,006	0,006	0,005	0,004	0,004	0,004	0,005	0,005	0,005	0,005	0,005																	
16. Cap10	0,006	0,005	0,005	0,006	0,006	0,005	0,004	0,004	0,004	0,005	0,005	0,005	0,005	0,005	0,000																
17. Cap15	0,008	0,004	0,001	0,007	0,006	0,007	0,005	0,005	0,005	0,005	0,005	0,005	0,005	0,005	0,005	0,005															
18. Cap16	0,007	0,003	0,000	0,006	0,006	0,007	0,005	0,003	0,005	0,004	0,004	0,004	0,004	0,005	0,005	0,005	0,001														
19. Cap18	0,007	0,003	0,000	0,006	0,006	0,007	0,005	0,003	0,005	0,004	0,004	0,004	0,004	0,005	0,005	0,005	0,001	0,000													
20. Cap19	0,007	0,003	0,000	0,006	0,006	0,007	0,005	0,003	0,005	0,004	0,004	0,004	0,004	0,005	0,005	0,005	0,001	0,000	0,000												
21. Cap20	0,007	0,003	0,000	0,006	0,006	0,007	0,005	0,003	0,005	0,004	0,004	0,004	0,004	0,005	0,005	0,005	0,001	0,000	0,000	0,000											
22. Cap21	0,007	0,003	0,002	0,006	0,005	0,006	0,005	0,004	0,005	0,003	0,005	0,005	0,005	0,004	0,004	0,004	0,002	0,002	0,002	0,002	0,002										
23. Cap22	0,007	0,003	0,000	0,006	0,006	0,007	0,005	0,003	0,005	0,004	0,004	0,004	0,004	0,005	0,005	0,005	0,001	0,000	0,000	0,000	0,000	0,002									
24. Cap23	0,007	0,003	0,000	0,006	0,006	0,007	0,005	0,003	0,005	0,004	0,004	0,004	0,004	0,005	0,005	0,005	0,001	0,000	0,000	0,000	0,000	0,002	0,000								
25. Cap24	0,007	0,003	0,000	0,006	0,006	0,007	0,005	0,003	0,005	0,004	0,004	0,004	0,004	0,005	0,005	0,005	0,001	0,000	0,000	0,000	0,000	0,002	0,000	0,000							
26. Cap25	0,007	0,003	0,000	0,006	0,006	0,007	0,005	0,003	0,005	0,004	0,004	0,004	0,004	0,005	0,005	0,005	0,001	0,000	0,000	0,000	0,000	0,002	0,000	0,000	0,000						
27. 874	0,007	0,002	0,001	0,006	0,006	0,007	0,004	0,003	0,004	0,004	0,004	0,004	0,004	0,005	0,004	0,004	0,002	0,001	0,001	0,001	0,001	0,002	0,001	0,001	0,001	0,001					
28. 6721	0,007	0,007	0,007	0,005	0,005	0,007	0,007	0,006	0,007	0,008	0,008	0,008	0,008	0,008	0,006	0,006	0,007	0,007	0,007	0,007	0,007	0,007	0,007	0,007	0,007	0,007	0,006				
29. 26909	0,002	0,005	0,007	0,005	0,006	0,009	0,008	0,007	0,008	0,009	0,009	0,009	0,009	0,009	0,007	0,007	0,008	0,007	0,007	0,007	0,007	0,007	0,007	0,007	0,007	0,007	0,007	0,008			
30. 26918	0,006	0,007	0,006	0,004	0,005	0,007	0,007	0,006	0,007	0,007	0,008	0,008	0,008	0,008	0,005	0,005	0,007	0,006	0,006	0,006	0,006	0,006	0,006	0,006	0,006	0,006	0,006	0,005	0,007		
31. 54731	0,003	0,006	0,007	0,003	0,003	0,007	0,008	0,006	0,008	0,008	0,008	0,008	0,008	0,008	0,006	0,006	0,007	0,007	0,007	0,007	0,007	0,006	0,007	0,007	0,007	0,007	0,007	0,005	0,004	0,004	
32. 68873	0,004	0,007	0,007	0,004	0,003	0,007	0,008	0,006	0,008	0,007	0,008	0,008	0,008	0,008	0,006	0,006	0,007	0,007	0,007	0,007	0,007	0,006	0,007	0,007	0,007	0,007	0,007	0,005	0,005	0,004	0,001
