# Supplementary material for: Antimicrobial susceptibility and multilocus sequence typing of Mycoplasma capricolum subsp. capricolum
Source: PLoS One. 2017 Mar 27;12(3):e0174700. doi: 10.1371/journal.pone.0174700 (PMC5367824; doi:10.1371/journal.pone.0174700)

S2 Fig.

Evolutionary tree based on *fusA* sequences. The tree was constructed using the neighbor-joining algorithm. Bootstrap percentage values were calculated from 1000 replications and values over 50% are displayed.

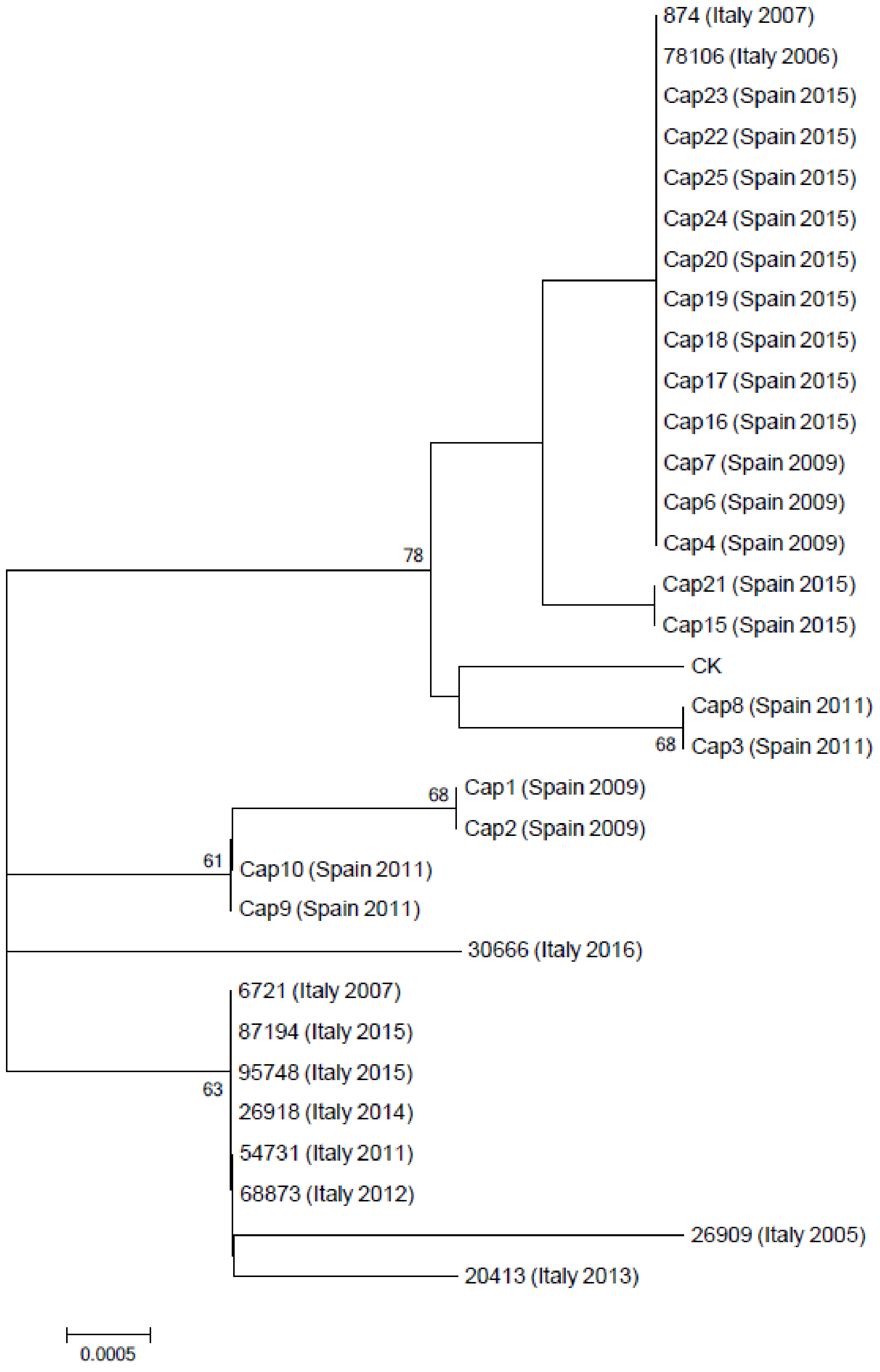

Supplement: S2 Fig — The tree was constructed using the neighbor-joining algorithm. Bootstrap percentage values were calculated from 1000 replications and values over 50% are displayed. (PDF) [file pone.0174700.s002.pdf]
