# Supplementary material for: Antimicrobial susceptibility and multilocus sequence typing of Mycoplasma capricolum subsp. capricolum
Source: PLoS One. 2017 Mar 27;12(3):e0174700. doi: 10.1371/journal.pone.0174700 (PMC5367824; doi:10.1371/journal.pone.0174700)
Supplement: S1 Table — (PDF) [file pone.0174700.s007.pdf]

S1 Table: GenBank accession numbers of sequences obtained used in this study

Strain	Genome	*fusA*	*glpQ*	*gyrB*	*lepA*	*rpoB*
**CK**	CP000123	-	-	-	-	-
**Cap1**	-	KU065166	KU065174	KU065182	KU065191	KU065201
**Cap2**	-	KU065166	KU065174	KU065182	KU065191	KU065201
**Cap3**	-	KU065167	KU065174	KU065183	KU065192	KU065202
**Cap4**	-	KU065168	KU065175	KU065182	KU065193	KU065203
**Cap6**	-	KU065168	KU065175	KU065182	KU065193	KU065203
**Cap7**	-	KU065168	KU065175	KU065182	KU065193	KU065203
**Cap8**	-	KU065167	KU065176	KU065183	KU065192	KU065202
**Cap9**	-	KU065169	KU065175	KU065184	KU065194	KU065204
**Cap10**	-	KU065169	KU065175	KU065184	KU065194	KU065204
**Cap15**	-	KU065170	KU065177	KU065185	KU065195	KU065205
**Cap16**	-	KU065168	KU065174	KU065186	KU065196	KU065205
**Cap17**	-	KU065168	KU065174	KU065186	KU065196	KU065205
**Cap18**	-	KU065168	KU065174	KU065186	KU065196	KU065205
**Cap19**	-	KU065168	KU065174	KU065186	KU065196	KU065205
**Cap20**	-	KU065168	KU065174	KU065186	KU065196	KU065205
**Cap21**	-	KU065170	KU065174	KU065187	KU065196	KU065206
**Cap22**	-	KU065168	KU065174	KU065186	KU065196	KU065205
**Cap23**	-	KU065168	KU065174	KU065186	KU065196	KU065205
**Cap24**	-	KU065168	KU065174	KU065186	KU065196	KU065205
**Cap25**	-	KU065168	KU065174	KU065186	KU065196	KU065205
**874**	-	KU065168	KU065174	KU065182	KU065196	KU065207
**6721**	-	KU065171	KU065178	KU065188	KU065197	KU065208
**26909**	-	KU065172	KU065179	KU065189	KU065198	KU065209
**20413**	-	KU065173	KU065180	KU065189	KU065199	KU065210
**78106**	-	KU065168	KU065181	KU065190	KU065200	KU065211
**87194**	-	KX423761	KX423767	KX423773	KX423779	KX423785
**95748**	-	KX423762	KX423768	KX423774	KX423780	KX423786
**30666**	-	KX423763	KX423769	KX423775	KX423781	KX423787
**26918**	-	KX423764	KX423770	KX423776	KX423782	KX423788
**54731**	-	KX423765	KX423771	KX423777	KX423783	KX423789
**68873**	-	KX423766	KX423772	KX423778	KX423784	KX423790
