# Supplementary material for: Antimicrobial susceptibility and multilocus sequence typing of Mycoplasma capricolum subsp. capricolum
Source: PLoS One. 2017 Mar 27;12(3):e0174700. doi: 10.1371/journal.pone.0174700 (PMC5367824; doi:10.1371/journal.pone.0174700)
Supplement: S2 Table — (PDF) [file pone.0174700.s008.pdf]

S2 Table: MIC results for each studied strain

Strain	Str	Kan	Gtm	Neo	Spt	Cip	Enr	Mrb	Dan	Mox	Nor	Dox	Til	Cli	Tlm	Spi	Lin	Eri
CK	>128	>128	>128	>128	>128	0,1	0,05	0,05	0,1	0,025	0,8	0,01	0,01	0,01	0,01	6,4	0,2	>12.8
Cap 1	>128	>128	>128	>128	>128	0,05	0,025	0,2	0,1	0,025	0,8	0,025	0,05	0,1	0,025	>12,8	0,8	>12.8
Cap 2	>128	>128	>128	>128	>128	0,1	0,1	0,2	0,1	0,05	0,8	0,025	0,1	0,1	0,01	>12,8	0,8	3,2
Cap 3	>128	>128	>128	>128	>128	0,05	0,1	0,1	0,1	0,025	0,4	0,01	0,05	0,01	0,025	3,2	0,2	>12,8
Cap 4	32	16	32	>128	16	0,2	0,1	0,2	0,1	0,05	0,8	0,025	0,05	0,01	0,025	>12,8	0,8	>12,8
Cap 6	32	32	64	>128	16	0,1	0,1	0,2	0,1	0,05	0,4	0,025	0,05	0,01	0,025	>12,8	0,4	>12,8
Cap 7	32	32	32	>128	16	0,1	0,1	0,2	0,1	0,025	0,8	0,025	0,05	0,01	0,025	>12,8	0,4	>12,8
Cap 8	32	16	32	>128	16	0,01	0,1	0,2	0,1	0,025	0,8	0,05	0,05	0,1	0,025	>12,8	0,4	>12,8
Cap 9**	>128	>128	>128	>128	>128	>12,8	0,2	0,4	0,4	0,05	0,4	0,1	0,1	>12,8	0,05	>12,8	>12,8	>12,8
Cap 10**	>128	>128	>128	>128	>128	>12,8	0,4	0,4	0,4	0,05	0,4	0,1	0,1	>12,8	0,05	>12,8	0,4	>12,8
Cap 15	64	32	64	>128	16	0,2	0,4	0,4	0,2	0,05	3,2	0,05	0,05	0,1	0,025	12,8	0,4	>12,8
Cap 16*	>128	32	64	>128	32	0,2	0,1	0,2	0,2	0,05	0,8	0,05	>12,8	>12,8	>12,8	>12,8	12,8	>12,8
Cap 17*	>128	64	64	>128	32	0,2	0,2	0,4	0,2	0,05	1,6	0,05	1,6	0,8	0,4	>12,8	3,2	>12,8
Cap 18*	>128	32	64	>128	32	0,2	0,2	0,4	0,2	0,05	1,6	0,025	3,2	0,8	0,8	>12,8	3,2	>12,8
Cap 19*	>128	32	64	>128	32	0,2	0,2	0,4	0,2	0,05	1,6	0,05	16	12,8	>128	>12,8	12,8	>12,8
Cap 20*	>128	32	32	>128	32	6,4	3,2	12,8	6,4	0,8	>12,8	0,025	0,1	0,2	0,05	>12,8	0,8	>12,8
Cap 21	64	32	64	>128	16	0,1	0,2	0,4	0,2	0,1	0,8	0,05	0,1	0,1	0,025	>12,8	0,4	>12,8
Cap 22*	>128	64	64	>128	32	0,2	0,1	0,4	0,2	0,1	0,4	0,025	64	1,6	128	>12,8	6,4	>12,8
Cap 23	>128	64	64	>128	32	1,6	3,2	1,6	3,2	0,4	>12,8	0,05	0,8	12,8	0,8	>12,8	>12,8	>12,8
Cap 24	>128	32	64	>128	32	0,1	0,1	0,2	0,1	<0,006	0,8	0,05	0,1	0,1	0,025	>12,8	0,4	>12,8
Cap 25*	>128	32	64	>128	32	0,1	0,2	0,4	0,1	0,1	0,8	0,05	16	16	16	>12,8	12,8	>12,8
874	16	16	>128	32	16	0,1	0,1	0,2	0,1	0,05	1,6	0,025	0,4	0,1	0,1	>12,8	0,8	>12,8
6721***	64	64	64	>128	32	0,1	0,2	0,4	0,1	0,1	0,8	0,2	0,1	0,4	0,025	>12,8	0,8	>12,8
26909	1,6	1,6	3,2	>128	1,6	0,2	0,2	0,4	0,2	0,1	1,6	0,05	0,1	0,4	0,025	>12,8	0,8	>12,8
20413	32	32	32	>128	16	0,2	0,2	0,4	0,2	0,1	1,6	0,05	0,1	0,2	0,025	>12,8	0,4	>12,8
78106***	>128	>128	>128	>128	>128	0,1	0,1	0,4	0,1	0,05	1,6	0,025	0,4	0,1	0,1	>12,8	0,8	>12,8
87194	>128	>128	>128	>128	32	0,2	0,2	0,2	0,1	0,025	0,8	0,05	0,1	0,8	0,025	>12,8	3,2	>12,8
95748	>128	>128	>128	>128	32	0,2	0,2	0,2	0,1	0,025	0,08	0,1	0,1	0,4	0,025	>12,8	0,4	>12,8
30666	32	64	64	>128	32	0,2	0,2	0,4	0,1	0,05	1,6	0,05	0,1	0,2	0,025	>12,8	0,8	>12,8
26918	32	32	64	>128	16	0,2	0,1	0,2	0,1	0,025	0,8	0,2	0,1	0,2	0,025	>12,8	0,4	>12,8
54731	32	32	64	>128	16	0,2	0,2	0,2	0,1	0,025	0,4	0,05	0,1	0,8	0,025	>12,8	3,2	>12,8
68873	16	32	64	>128	32	0,2	0,2	0,2	0,1	0,025	0,8	0,05	0,1	0,8	0,025	>12,8	3,2	>12,8

*, **, ***: Isolates from the same herd at different time points
